# Enteric Fever Complicated by Intestinal Perforation in Children: A Persistent Health Problem Requiring Surgical Management

**DOI:** 10.12669/pjms.36.5.2270

**Published:** 2020

**Authors:** Muhammad Azhar, Naima Zamir, Mishraz Shaikh, Inayat ullah

**Affiliations:** 1 Muhammad Azhar, FCPS, Senior Registrar, Department of Paediatric Surgery, National Institute of child Health, Karachi, Pakistan; 2 Naima Zamir, FCPS, FACS, Associate Professor, Department of Paediatric Surgery, National Institute of child Health, Karachi, Pakistan; 3 Mishraz Shaikh, FCPS, Senior Registrar, Department of Paediatric Surgery, National Institute of child Health, Karachi, Pakistan; 4 Inayat ullah, Resident Pediatric Surgery, Department of Paediatric Surgery, National Institute of child Health, Karachi, Pakistan

**Keywords:** Enteric perforation, Ileostomy, Tube laparostomy

## Abstract

**Objective:**

To evaluate clinical presentation and surgical outcome in children with enteric perforation.

**Methods:**

A descriptive retrospective study was conducted in Department of Paediatric Surgery at National Institute of Child Health, Karachi from August 2016 and September 2019, in children 12 years of age and under with diagnosis of enteric perforation. Data about age, gender, duration of illness, hemodynamic status and baseline investigation on admission was reviewed. Details about patients operated early and those who required prolong resuscitation and were operated after 24 hours of admission, need for tube laparostomy, operative findings, type of surgical procedure performed and post-operative outcome were reviewed. Data was analyzed using SPSS version 22.

**Results:**

Ninety-seven patients, 60(61.85%) males and 37(38.14%) females were managed during the study period with age ranged from 3-12 years (mean 7.82, ± 2.94 years).and duration of symptoms ranged from 7-30 days (mean 15.56, ± 9.39days). High grade fever and abdominal pain were seen in all patients (100%). Pneumoperitoneum was noted in 71(73.19%) cases on X-ray abdomen. Fifty-one (52.57%) children were anemic and required blood transfusion before surgery. Seventy-one (73.19) patients were optimized and operated within 24 hours while 28(28.86%) cases required more resuscitation so tube laparostomy was done initially and operated after 24 hours. Seventy nine (81.44%) cases had single perforation, 14(14.43%) cases had multiple and four had sealed perforation. Primary repair of perforation was done in 37(38.14%) cases, while ileostomy in 65(76.01%) cases. Postoperatively wound infection was seen in 71(73.19%) cases, intra-abdominal collections in 31(31.95%) and burst abdomen in nine (9.27%) cases. Overall mortality was 12.37%. Till date in 47 patients (72.30%) reversal of stoma has been done.

**Conclusions:**

Enteric perforation in children presents usually with hemodynamic instability and sepsis due to prolong period of illness. Therefore, regardless of surgical procedure performed it is associated with high morbidity and mortality.

## INTRODUCTION

Enteric fever remains a health concern in third-world and more recently the emergence of drug resistant strains of Salmonella Typhi has challenged diagnostic, and treatment capabilities of existing health system.^1^ Despite global efforts at its eradication it is still prevalent due to lack of access to clean drinking water, unhygienic living conditions and poor health care delivery systems.^2^,^3^ Children are common target of this condition.^4^,^5^ Although Enteric fever is a medical condition but if left untreated can have a devastating outcome in the form of intestinal perforation.^6^ Prolong period of febrile illness followed by complications of intestinal perforation are causes of high morbidity and mortality.^7^ Enteric perforations pose a great challenge for surgeons to manage. The current study was conducted to evaluate clinical presentation and surgical outcome in children with enteric perforation

## METHODS

This was a retrospective study of children aged 12 years and under with clinical diagnosis of enteric perforation treated at the department of Paediatric Surgery, National institute of child Health, from August 2016 and September 2019.

After ethical approval by institutional review board (Ref no. IERB 40/2019) medical record was reviewed to identify all cases based upon history of high grade fever for more than one week followed by signs of peritonitis, and per-operative findings of distal ileal perforation at its anti-mesenteric border. All cases of ileal perforations due to trauma, tuberculosis, volvulus of gut, etc. were excluded.

The study included details regarding age, gender, duration of symptoms and hemodynamic status of patients at the time of admission. Information regarding initial complete blood count, electrolytes, BUN, serum creatinine levels and presence of free air on X-ray abdomen was retrieved. Data about patients who required resuscitation, transfusion of blood and its products, correction of electrolyte and need for a tube laparostomy was reviewed. According to institutional protocol patients with hemodynamic instability or those in sepsis requiring prolong resuscitation, underwent tube laparostomy as a bridging procedure. Additionally data regarding operative findings, surgical procedure performed either primary repair of the perforation or exteriorization of perforation (Ileostomy) and post-operative complications was also reviewed. The patients in whom operative finding showed extensive peritoneal contamination, friable and inflamed gut, ileostomy was made. In other cases where there was limited contamination and gut otherwise was healthy and less friable, primary repair was done. Data was analyzed using SPSS version 22.

## RESULTS

A total of 97 patients were managed during the study period. There were 60 (61.85%) males and 37 (38.14%) females, with age ranged from 3-12 year (mean 7.82, ± 2.94 years) and duration of symptoms ranged from 5-30days (mean 15.56, ± 9.39 days). High grade fever followed by abdominal pain was the most consistent finding seen in all cases (100%). Other clinical features are shown in Table-I.

**Table-I:** Clinical features on admission.

Clinical features	Number of patients (N=97)	Percent
***Symptoms***		
Abdominal pain	97	100%
Fever	97	100%
Abdominal distension	63	64.94%
Vomiting	37	38.14%
Diarrhea	25	25.77%
Constipation	11	11.34%
Altered sensorium	3	3.09%

Three patients also presented with altered sensorium. Fifty-one (52.50%) patients were anemic and required transfusion and twenty-six patients required platelets transfusion before surgery as well. There were 19 (19.85%) cases with electrolyte imbalance on presentation. Sub diaphragmatic free air was noted in 71 (73.19%) cases on x-ray abdomen. Seventy-one (73.19%) patients were optimized within 24 hours and underwent laparotomy. In 26 cases tube laparostomy was done followed by surgery after 24 hours. In 79 (81.44%) cases single and 14 (14.43%) had multiple perforations, while four were with sealed perforation.

Primary repair of perforation was done in 28 (28.86%) patients. Among them16(57.14%) had wound infection, three developed fecal fistula out of which two died of sepsis, one had burst abdomen due to leak and ileostomy was added. This patient also died later. Five patients were re-admitted with abdominal pain and unsettling fever. They were found to have inter-loop collections and responded to non-operative management.

In this study ileostomy was made in 65 (67.01%) patients, as a first procedure Among them 55 (84.61%) cases developed post-operative wound infection and variable wound dehiscence. There were 31 (47.69%) cases of intra-abdominal collection and ten cases of burst abdomen. In burst abdomen group, eight patients were re-operated and closure done, but in two cases only skin closure was possible.

Eight patients who initially had tube laparostomy followed by stoma formation, died of sepsis. One patient died following discharge from hospital. A total of 12 (12.37%) patients expired in this series, eight with initial ileostomy, and three with primary repair group. It is important to mention that more than one complication occurred in patients. Till date in forty seven patients (72.30%) reversal of stoma has been performed after four to six months of initial surgery. (Summary of management Algorithm is shown below).

## DISCUSSION

Intestinal perforation, a serious complication of enteric fever is seen quite commonly in our part of world. This may be due to delay in diagnosis or inappropriate treatment.^6^ In current study this grievous complication was seen more common in male population. The plausible explanation to this male dominance may be that go out of homes more frequently and eat and drink as compared to females. However, Khan M et al proposed that genetic and host inflammatory response also play a role.^8^ High grade fever followed by abdominal pain was the most consistent feature as also seen in various studies.^5^ Three patients presented with psychosis. Neurological symptoms although uncommon in children, occur in 5%-35% cases in the form of delirium, dystonia, hyperreflexia and clonus.^9^


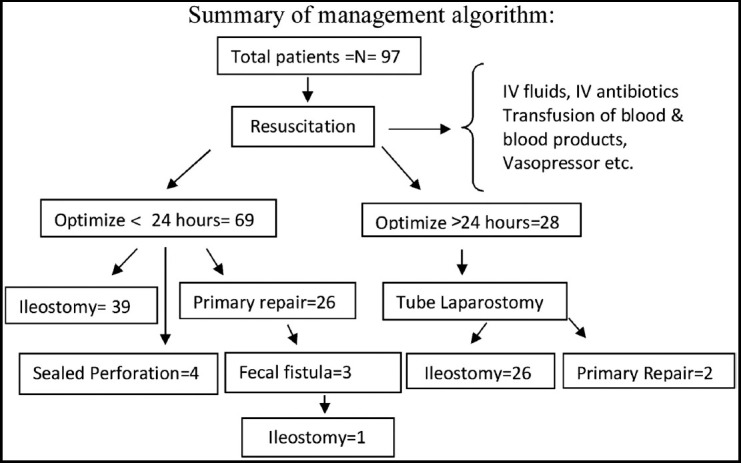


Enteric perforations pose a great surgical challenge to manage due to its pre and post -operative consequences. These patients may present with hemodynamic instability, electrolyte imbalance, anemia and sepsis.^10^ The surgery must be delayed till adequate optimization is achieved in regards to fluid and electrolyte balance, correction of haemoglobin, platelets and achieving urine ouput of more than 1ml/kg/hour.^11^ There were 52.50% patients with below optimal haemoglobin who required blood transfusion, and 26 cases required platelets transfusion before surgery. Tube laparotomy - a bridging procedure before surgery, in patients who need prolong optimization to reduce the septic load and elevated intra-abdominal pressure,^12^,^13^ was done in 28% of patients.

Primary closure always attract surgeons but it is not always possible because of many challenges.^14^ Since majority of patients in current study had prolong duration of illness, compromised clinical status and heavy peritoneal contamination, primary repair was possible only in 28.86% cases. Studies have shown that bowel in enteric fever is inflamed, does not hold sutures and cut through easily. Freshening of margins increases the size of perforation and layered closure is even more difficult. Resection anastomosis carries high risk of disruption. Exteriorization of perforation or repair and proximal ileostomy, are more appropriate in such situation.^15^,^16^ This was done in 67% of patients in our series.

Postoperative complications lead to prolong morbidity and mortality.^17^ In this series, 71 (73.19%) cases developed wound infection and variable forms of wound dehiscence which were managed conservatively. Studies show that infected wound healed spontaneously after removing some sutures to permit exit of purulent discharge followed by daily dressing.^18^ Burst abdomen is of critical importance and can be a complication of intra-abdominal collection or leak of repaired gut, apart from poor healing capacity. Despite adequate irrigation of abdominal cavity and placement of drain, infected foci lead to collection. It is therefore important to explore burst abdomen thoroughly, look for any leaks and drain collections at secondary surgery. Burst abdomen occurred in 11 cases (11.34%) out of which ten case had intra-abdominal collections and one had leak.

Addition of stoma is one of the biggest morbidity that a patient has to deal with. Wound infection in early postoperative period can be secondary to stoma contaminating the wound. Later loss of enzyme rich and hyperosmolar fluid from stoma leads to peristomal skin excoriation, fluid and electrolyte imbalance and failure to thrive.^19^ Stoma bag management can be challenging in small children. Parents need to be vigilant to empty the bag frequently to prevent overflow and dislodgement from its base due to increased pressure within the bag. Due to high cost of stoma bag and issues related to its care, some parents tend to keep piece of cloth over stoma.^20^ This further adds to morbidity. Keeping above facts in view, early reversal is advised. Mortality was high, seen in 11(11.34%) patients with sepsis being the cause, except for one patient who died due to fluid and electrolyte loss.

### Limitations of study

Some of the patients with suspected enteric perforation expired before surgical intervention and were not included in the study so the actual number could not be documented. Non availability of blood and tissue culture results was also a limitation, as diagnosis was made on clinical ground only.

## CONCLUSION

Initial control of sepsis and achieving hemodynamic instability is the key in management of children with enteric perforation before any surgical intervention. It still carries a high rate of mortality and morbidity regardless of surgical procedure performed. This calls for early and adequate treatment of enteric fever to prevent its life threatening consequences.

### Author’s contribution

**MA & NZ:** Conceived the idea and designed the study, are responsible for integrity of study.

**MS:** was involved in data collection. **I:** was involved in data analysis and data interpretation..
